# Iron metabolism-related genes reveal predictive value of acute coronary syndrome

**DOI:** 10.3389/fphar.2022.1040845

**Published:** 2022-10-18

**Authors:** Cong Xu, Wanyang Li, Tangzhiming Li, Jie Yuan, Xinli Pang, Tao Liu, Benhui Liang, Lixin Cheng, Xin Sun, Shaohong Dong

**Affiliations:** ^1^ Shenzhen People’s Hospital, First Affiliated Hospital of Southern University of Science and Technology, Second Clinical Medicine College of Jinan University, Shenzhen, China; ^2^ School of Mathematics, South China University of Technology, Guangzhou, China; ^3^ International Digital Economy Academy, Shenzhen, China; ^4^ Department of Cardiology, Xiangya Hospital, Central South University, Changsha, China

**Keywords:** acute coronary syndrome, iron metabolism, transcriptome, prediction model, diagnosis

## Abstract

Iron deficiency has detrimental effects in patients with acute coronary syndrome (ACS), which is a common nutritional disorder and inflammation-related disease affects up to one-third people worldwide. However, the specific role of iron metabolism in ACS progression is opaque. In this study, we construct an iron metabolism-related genes (IMRGs) based molecular signature of ACS and to identify novel iron metabolism gene markers for early stage of ACS. The IMRGs were mainly collected from Molecular Signatures Database (mSigDB) and two relevant studies. Two blood transcriptome datasets GSE61144 and GSE60993 were used for constructing the prediction model of ACS. After differential analysis, 22 IMRGs were differentially expressed and defined as DEIGs in the training set. Then, the 22 DEIGs were trained by the Elastic Net to build the prediction model. Five genes, PADI4, HLA-DQA1, LCN2, CD7, and VNN1, were determined using multiple Elastic Net calculations and retained to obtain the optimal performance. Finally, the generated model iron metabolism-related gene signature (imSig) was assessed by the validation set GSE60993 using a series of evaluation measurements. Compared with other machine learning methods, the performance of imSig using Elastic Net was superior in the validation set. Elastic Net consistently scores the higher than Lasso and Logistic regression in the validation set in terms of ROC, PRC, Sensitivity, and Specificity. The prediction model based on iron metabolism-related genes may assist in ACS early diagnosis.

## Introduction

Iron deficiency has detrimental effects in patients with acute coronary syndrome (ACS) (Kang et al., 2012; Das De et al., 2015), which is a common nutritional disorder affects up to one-third people worldwide (Stoltzfus, 2001). Apart from erythropoiesis, iron serves in many fundamental and physiological processes, such as oxygen transport and storage, mitochondrial function, and immune system (Crielaard et al., 2017). Populations at high risk of iron deficiency often includes infants, children, adolescents, elderly persons and women, while young adults ranging from 30 to 45 years old usually have normal iron levels.

Although iron status has been implicated in cardiovascular disease (CVD), the underlying mechanism of expression regulation is still unclear. Patients with high iron stores tend to have high risk in ACS (Jiang et al., 2004), such as those who had recently undergone a myocardial infarction, disodium EDTA chelation of heavy metals decreased adverse cardiovascular outcomes (Lamas et al., 2013). These observations contrast with the results that a higher iron status may have a preventive impact on the risk of coronary heart disease, according to a meta-analysis of observational studies (Das De et al., 2015). Given the growing prevalence, high morbidity and mortality burden, ACS was taken as the most crucial challenge in contemporary cardiology. Despite treatment improvement and risk factor reduction, young patients with coronary artery disease (CAD) remain at high risk of acute cardiovascular events (Tsao et al., 2022). The role of iron deficiency in the progression of coronary disease for young patients has not been well recognized.

A growing body of evidences have demonstrated that patients with CAD are more likely suffered from iron deficiency (Kang et al., 2012; Ponikowska et al., 2013; Grammer et al., 2014; Das De et al., 2015; Jankowska et al., 2015). Lacking of iron may impair immune response, myocardial cell metabolism and oxidative stress, which is associate with coronary dysfunction. However, the specific role of iron in CAD progression is opaque. Exploring mechanisms behind iron discrepancy in CAD may promote the understanding of coronary disease pathophysiology.

Recent years, biotechnological advancements have enabled researchers to produce and analyze molecular datasets, such as genomics (Wu et al., 2021), coding transcriptomics (Cheng et al., 2020; Yang et al., 2021), non-coding transcriptomics (Zheng et al., 2021a; Song et al., 2021; Li et al., 2022), proteomics (Li et al., 2020), epigenomics (Zheng et al., 2021b; Wu et al., 2022), metabolomics (Liu et al., 2020a), and single cell data (Wang et al., 2022). Simultaneously, the impact of applications of machine learning algorithms to Invasive Diagnosis (IVD) have been well documented across a large number of diseases (Wang et al., 2020a; Wang et al., 2020b).

The impact of iron deficiency on the occurrence and development of young ACS has not been verified. Therefore, we sought to investigate the relationship of iron metabolism related genes (IMRGs) and ACS. In this study, we constructed a prediction model for ACS diagnosis using the transcriptome data of iron metabolism related genes. The performance of the generated diagnostic model was superior to the other methods.

## Methods

### Iron metabolism related genes

The iron metabolism related genes (IMRGs) used in this study were mainly collected from Molecular Signatures Database v7.5.1 (http://www.gsea-msigdb.org/) (Subramanian et al., 2005), consisting of 51 datasets, as well as two relevant literature (Mou et al., 2020; Zhang et al., 2020). In total, 1,239 unique IMRGs were finally obtained by removing duplicated genes.

### Expression datasets and data processing

Gene expression datasets were downloaded from the Gene Expression Omnibus (GEO, http://www.ncbi.nlm.nih.gov/geo/) database (Barrett et al., 2013). Two datasets GSE61144 and GSE60993, detected by platforms of Sentrix Human-6 v2 Expression BeadChip (GPL6106) and Illumina HumanWG-6 v3.0 expression beadchip (GPL6884), respectively, were used in this study. The two platforms were applied for the mapping between probe name and gene name. Genes with duplicate probe names were averaged in expression value, resulting in 929 IMRGs for GSE60993 and 949 IMRGs for GSE61144, respectively. Both gene expression datasets were log2 transformed and quantile normalized (Cheng et al., 2016a; Cheng et al., 2016b; Liu et al., 2019).

GSE61144 contains 14 ACS patients and 10 normal samples, which is used as a discovery set divided into 75% as the training set and 25% as the test set. GSE60993 contains 26 ACS patients and seven normal samples and it is used as a cross-platform validation set.

### Differential analysis

Differentially expressed genes (DEGs) between normal samples and patients with acute coronary syndrome (ACS) were analyzed using the built-in functions of the scipy library of python. DEGs were defined as those genes with Wilcoxon Rank-Sum *p*-value<0.05 and $\left|\log_{2}\left(fold\;change\right)\right|$ >1.5. In the training set, 22 out of the 929 IMRGs were identified as DEGs, including CD7, CYP4F3, DAPK2, DUSP1, FKBP5, G6PD, HLA-DQA1, IL13RA1, ITGAM, LCN2, LILRB3, LTF, MXD1, NARF, ORM1, PADI4, PRKCH, PYHIN1, SLC11A1, TNFAIP6, UCP2 and VNN1. All these genes were also detected in the validation set GSE60993, and their corresponding gene expression values are available.

### Model construction

We constructed the diagnostic signature using Elastic-Net Regression (EN), which is a linear regression model trained using L1 and L2 norm as prior regularization terms. This combination allows fitting to a model where only a few parameters are non-zero sparse, like Lasso, but it still maintains some Ridge-like regularity properties.

The label is added to the data sets GSE61144 and GSE60993 as the dependent variable y, with value of (HPS2-THRIVE randomized placebo, 2013), where 0 represents the normal group and one represents the ACS group. The expression value of the 22 DEGs were used as the independent variable X. The GSE61144 dataset is divided into 75% as the training set to train the model. After the optimal parameters of elastic network were determined using multiple trainings, the regularization parameter *α* is set to 0.01 and the parameter l1_ratio is set to 0.7. When l1_ratio = 0, the penalty is L2 norm. When l1_ratio = 1, it is L1 norm. When 0 < *l*1_ratio <1, the penalty is the combination of L1 and L2.

The output y is a continuous value ranging from 0 to 1, which cannot directly determine the status of a patient, i.e., ACS or normal. Therefore, a label discrimination threshold C is set. When the predicted value of y is greater than C, it is determined that y is 1, otherwise y is 0, where one indicates ACS and 0 represents normal. The selection of the label discrimination threshold C is based on the test set, which is composed of the same number of samples with label 0 and 1.

$$C=mean\;\left({pred}_{test}\right)$$ (1)

where ${pred}_{test}$ is the prediction result of the test set, and C is the mean of the prediction result of the test set.

Due to the randomness of the model training process, in order to eliminate the effect of fluctuations in the results caused by randomness, we used the elastic network to randomly test 10,000 times. Screen the results with a ROC score greater than 85% on the validation set, and then take the eigengenes i whose frequency $P_{i}$ is greater than 75%. Five genes were finally screened, including CD7, HLA-DQA1, LCN2, PADI4, and VNN1.

$$P_{i}=\frac{n_{i}}{N}$$ (2)

where N is the number of results with ROC greater than 85%, and $n_{i}$ is the number of times the differential genes i appears in these N results.

Finally, we put the five IMRGs into the elastic network to retrain a new model. The iron metabolism related gene signature (imSig) is shown as below,

y=−0.19826267∗(CD7)−0.52581069∗(HLA−DQA1)+0.47276387∗(LCN2)+0.73707807∗(PADI4)−0.18809673∗(VNN1) (3)

where gene PADI4 has the largest weight. The determinant function is

$$y=\left\{ \begin{array}{c} 0\;,\;y<C\\ 1\;,\;y\geq C \end{array}\right.\;\left(C=0.35\right)$$ (4)

## Results

### Study population

**FIGURE 1:**
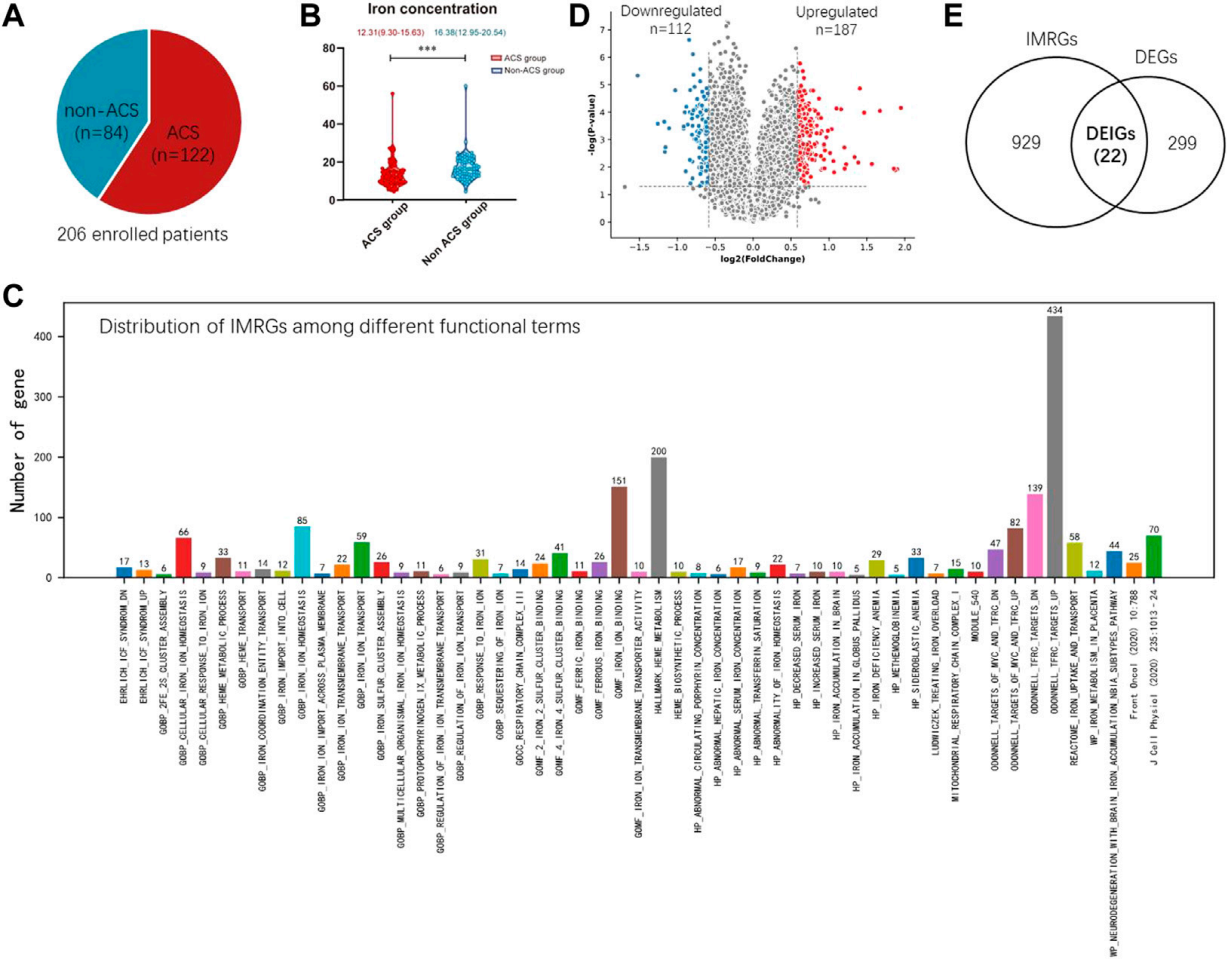
Establishment of IMRGs. **(A)** Composition of enrolled samples. **(B)** Boxplot showing the iron difference clinically. **(C)** Identification of DEGs from the training set GSE61144. **(D)** Venn diagram of all DEGs and IMRGs. **(E)** Distribution of iron metabolism-related genes among different functional terms.

This study (Multi-omics Study of Young Adults Coronary Syndrome Patients, Young-COSMOS, http://www.clinicaltrials.gov/NCT04864457) was a single center, prospective, open-label, case-crossover clinical trial, that recruited participants between November 2020 and December 2021 in Shenzhen people’s hospital. We enrolled 206 young adults aged from 30 to 45 years old with chest pain manifestation (Figure 1A), patients were eligible for participation in the trial if they had chest pain manifestation, and requested to experience medical history obtaining, physical examination, laboratory analysis, electrocardiogram monitoring, transthoracic echocardiography as well as coronary angiography. Taken this information together, they were divided to ACS group and non-ACS group. Exclusion criteria were myocardiopathy, myocarditis, cerebral infraction, connective tissue diseases, estimated glomerular filtration rate of less than 20 ml/min per 1·73 m^2^, New York Heart Association class III or IV heart failure or left ventricular ejection fraction of less than 30%, history of mental disorder or malignant tumor, and elevation of creatine kinase more than five times above normal or hepatic aminotransferase more than three times above normal. All patients provided written informed consent. The protocol was approved by ethics committee of Shenzhen people’s hospital.

Importantly, we found the ACS group has a significant low serum iron concentration than the non-ACS group (*p* < 0.001, Wilcoxon test, Figure 1B). In the non-ACS group, the average iron concentration is 16.38 (12.95–20.54), whereas this value is 12.31 (9.30–15.63) in the ACS group. These results motivated us to investigate whether the iron-related genes are ACS biomarkers and their expression abundance may indicate ACS diagnosis.

### Establishment of iron metabolism-related genes

The iron metabolism related genes (IMRGs) were mainly collected from Molecular Signatures Database (mSigDB v7.5.1) (http://www.gsea-msigdb.org/) (Subramanian et al., 2005) and two relevant studies [ (Zhang et al., 2020), (Mou et al., 2020)]. mSigDB consists of 51 functional gene sets describing IMRGs (Figure 1C). Gene set ODONNELL TFRC_TARGETS_UP contains the most IMRGs 434) that are up-regulated in P493-6 cells upon knockdown of transferrin receptor 1 (TFRC1) by RNAi. TFRC1 is a major mediator of iron uptake in mammalian cells and its overexpression is a common feature of human malignancies [ (Corral et al., 2021)]. The second largest gene set is HALLMARK_HEME_METABOLISM, which includes 200 genes involved in metabolism of heme, a cofactor consisting of iron and porphyrin. The minimal gene set is HP_METHEMOGLOBINEMIA, which describes abnormally increased levels of methemoglobin in the blood. There is an oxidized ferric iron (Fe+3) rather than the reduced ferrous form (Fe2+) that is normally found in this form of hemoglobin. Methemoglobin has a reduced affinity for oxygen, resulting in a reduced ability to release oxygen to tissues. 95 other IMRGs were collected from two recently published works by Zhang et al. and Mou et al. [(Zhang et al., 2020), (Mou et al., 2020)]. The distribution of IMRGs among different functional gene sets are shown in Figure 1C. In total, 1,239 unique IMRGs were finally obtained by removing duplicated genes.

### Differential and functional analysis of IMRGs

Although the Young-COSMOS study is in process, we motivated to take a quick glance at the predictive value of the IMRGs in ACS diagnosis. Two publicly available blood transcriptome datasets collected from GEO database (GSE61144 and GSE60993) were used for constructing the prediction model of acute coronary syndrome (ACS). GSE61144 contains peripheral blood samples from 14 patients with STEMI and 10 normal controls. GSE60993 includes 26 blood samples of ACS patients covering three subtypes, ST-elevation myocardial infarction (STEMI, n = 7), non-ST-elevation MI (NSTEMI, n = 10) and unstable angina (UA, n = 9), as well as seven normal controls. For GSE61144, we identified 187 up-regulated and 112 down-regulated differentially expressed genes (DEGs) with Fold Change (FC) > 1.5 and *p*-value < 0.05 (Wilcoxon test, Figure 1D).

**FIGURE 2:**
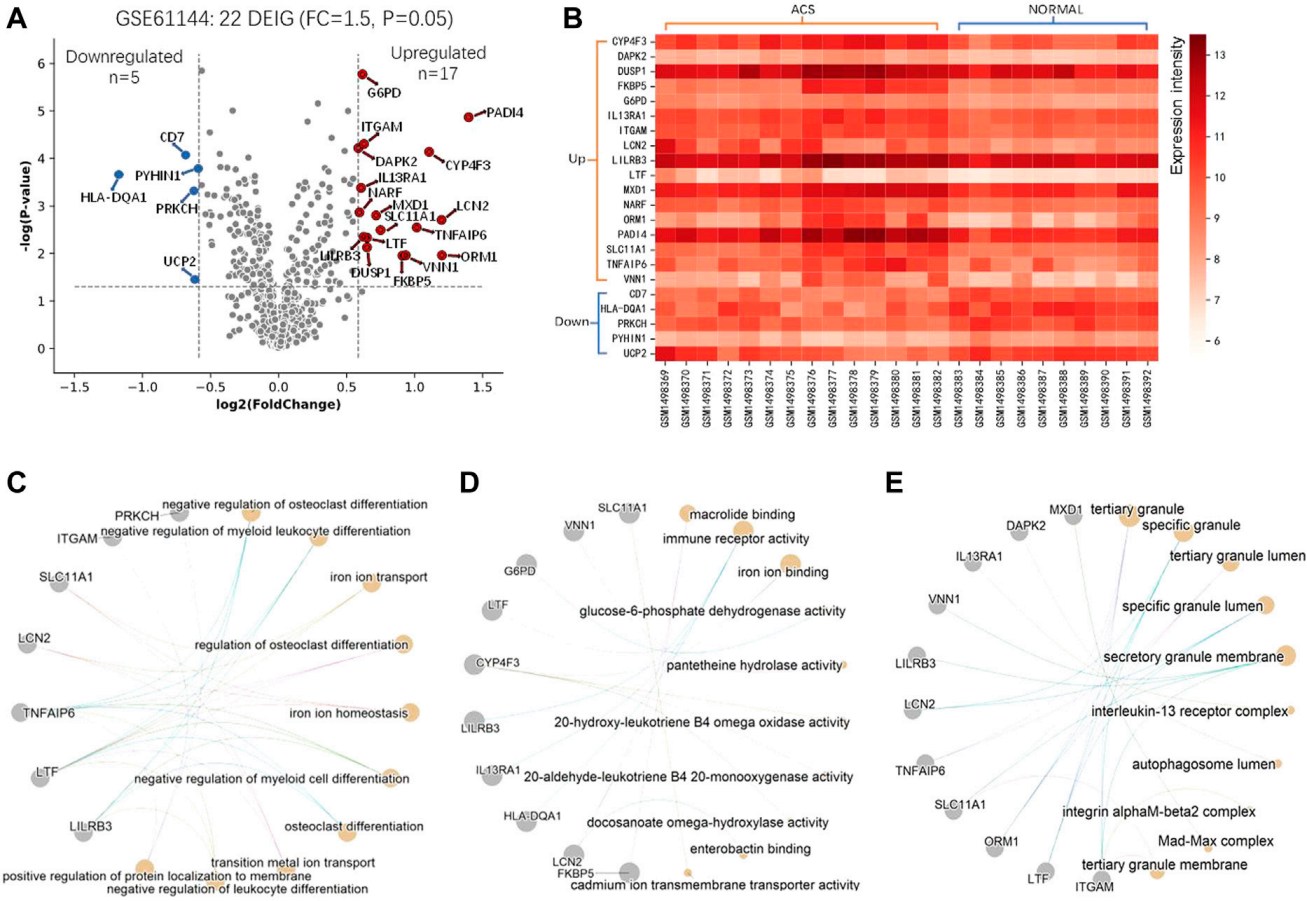
Differential and functional analysis of DEIGs. **(A)** Identification of differentially expressed IMRGs. **(B)** Heatmap of the identified DEIGs. Enriched functions of the DEIGs in the ontology of Biological Process **(C)**, Molecular Function **(D)**, and Cell component **(E)**.

For these DEGs, 22 out of them are IMRGs in the data set GSE61144, which serves as the training set in the next section (Figure 1E). We defined these differentially expressed IMRGs as DEIGs, among which 17 are up-regulated and five were down-regulated (Figures 2A,B). DEIGs were mainly involved in biological processes of iron ion transport and homeostasis, osteoclast differentiation, regulation of protein localization to membrane, *etc.* (Figure 2C). For molecular functions, DEIGs were enriched in terms including iron ion binding, macrolide binding, immune receptor activity, *etc.* (Figure 2D). As expected, these DEIGs tend to locate in lumens and membranes, such as granule and autophagosome lumen, secretory and tertiary granule membrane, *etc.* (Figure 2E).

### Construction of imSig

**FIGURE 3:**
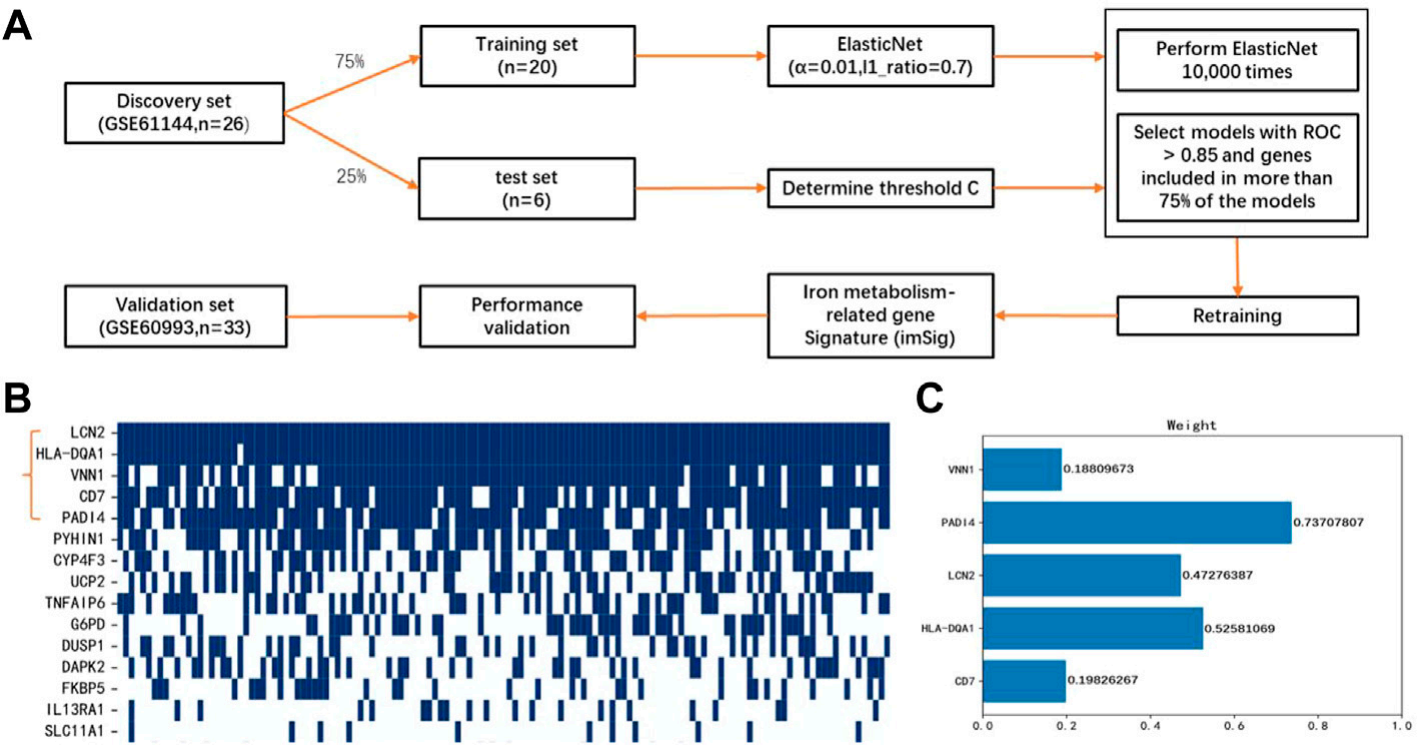
Construction of miSig model **(A)** Workflow of this study. **(B)** Feature gene selection using 10,000 random models. Blue grid indicates the gene is included in a powerful model with ROC >0.85, while white grid represents the gene is not included in any powerful model. **(C)** Weight of genes in the miSig model.

We aimed to determine an IMRG-based molecular signature of ACS and to identify novel serum iron gene markers for early stage of ACS. To this end, GSE61144 was used as the discovery set, which was divided to 75% for model training and 25% for parameter optimization (Figure 3A). The 22 DEIGs were trained by the Elastic Net to build the prediction model. Since the resulting genes were not consistent when using different random computation seeds, we performed LASSO 10,000 times and selected the genes occurred most frequently. A majority of the genes were randomly picked up and only five genes were consistently identified in more than 75% (>7,500) rounds with ROCs greater than 0.85 (Figure 3B), indicating the importance and generalizability of these genes in classification. The weights of the five genes, PADI4, HLA-DQA1, LCN2, CD7, and VNN1, were then retained to obtain the optimal performance (Figure 3C). The details of these genes are listed in Table 1. Finally, the generated model was assessed by the validation set GSE60993 using a series of evaluation measurements.

**TABLE 1:** Determined genes in imSig.

Gene symbol	Gene name	Entrez ID	Weight	Terms
PADI4	Peptidyl Arginine Deiminase 4	23,569	0.7371	ODONNELL_TFRC_TARGETS_UP
HLA-DQA1	Major Histocompatibility Complex, Class II, DQ Alpha 1	3,117	0.5258	HP_IRON_DEFICIENCY_ANEMIA
				ODONNELL_TARGETS_OF_MYC_AND_TFRC_UP
				ODONNELL_TFRC_TARGETS_UP
LCN2	Lipocalin 2	3,934	0.4728	GOBP_CELLULAR_IRON_ION_HOMEOSTASIS
				GOBP_IRON_COORDINATION_ENTITY_TRANSPORT
				GOBP_IRON_ION_HOMEOSTASIS
CD7	CD7 Molecule	924	0.1983	GOBP_IRON_ION_TRANSPORT
				GOBP_SEQUESTERING_OF_IRON_ION
VNN1	Vanin 1	8,876	0.1881	GOMF_IRON_ION_BINDING
				LUDWICZEK_TREATING_IRON_OVERLOAD
				REACTOME_IRON_UPTAKE_AND_TRANSPORT

Among the five imSig genes, PADI4 contributes the most in the model. It is a member of a gene family which encodes enzymes responsible for the conversion of arginine residues to citrulline residues. PADI4 may play a role in granulocyte and macrophage development leading to inflammation and immune response, both of which are the main pathogenesis of ACS.

### Performance evaluation of imSig

**FIGURE 4:**
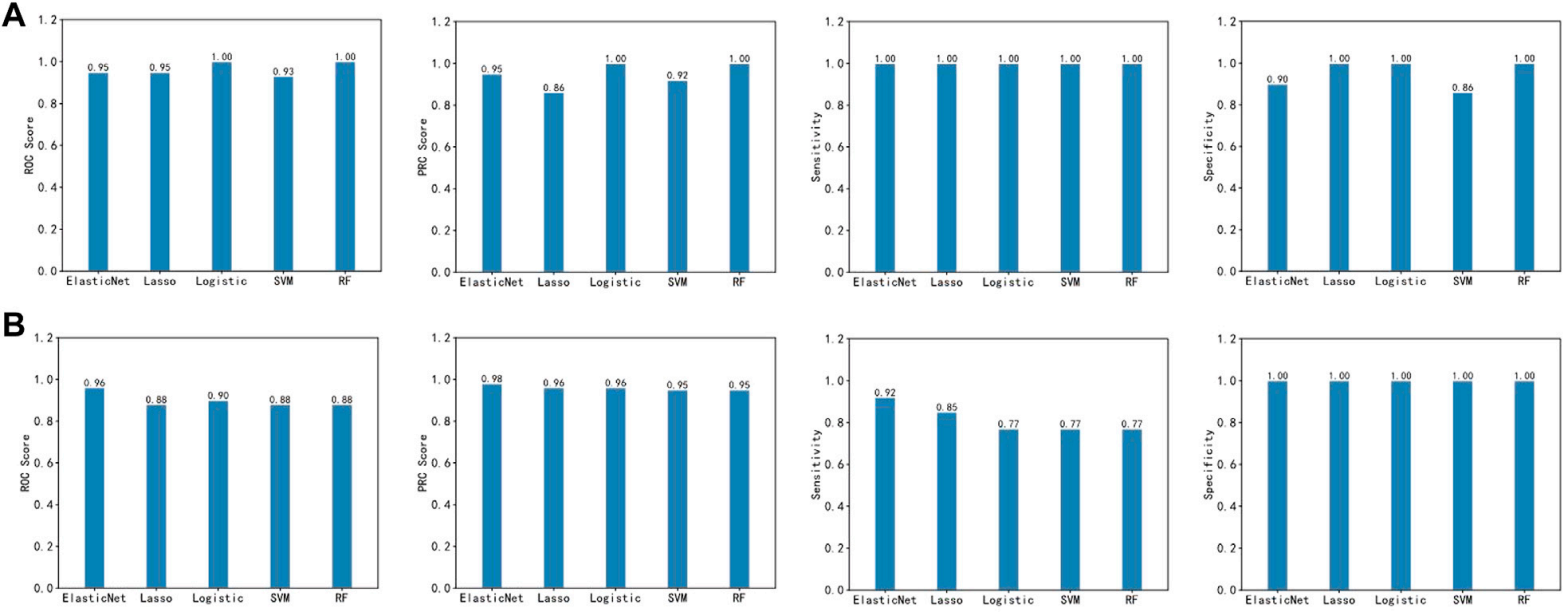
Prediction performance of the five machine learning methods in the training set **(A)** and the validation set **(B)**.

The final imSig5 model using Elastic Net obtained a ROC of 0.95 on the training set and 0.96 on the cross-platform validation set. Then, we compared the performance of Elastic Net with other two machine learning methods, Lasso and Logistic regression. In the validation set, the performance of Elastic Net was superior to the others (0.88 for Lasso and 0.90 for Logistic regression, Figure 4). To perform a comprehensive evaluation, we also compared the performance of the three models using PRC, Sensitivity, and Specificity. Elastic Net consistently scores the higher than Lasso and Logistic regression in the validation set. Specifically, the PRCs are 0.98, 0.96, and 0.96 for Elastic Net, Lasso and Logistic regression, respectively. Although all the three methods achieved a specificity of 1, Elastic Net obtained a sensitivity of 0.92, which is much higher than Lasso (0.85) and Logistic regression (0.77). Apart from Lasso and Logistic regression, we also compared the performance of Elastic Net with two other machine learning methods, Random Forest (RF) and Support Vector Machine (SVM), and achieved the same conclusion.

**FIGURE 5:**
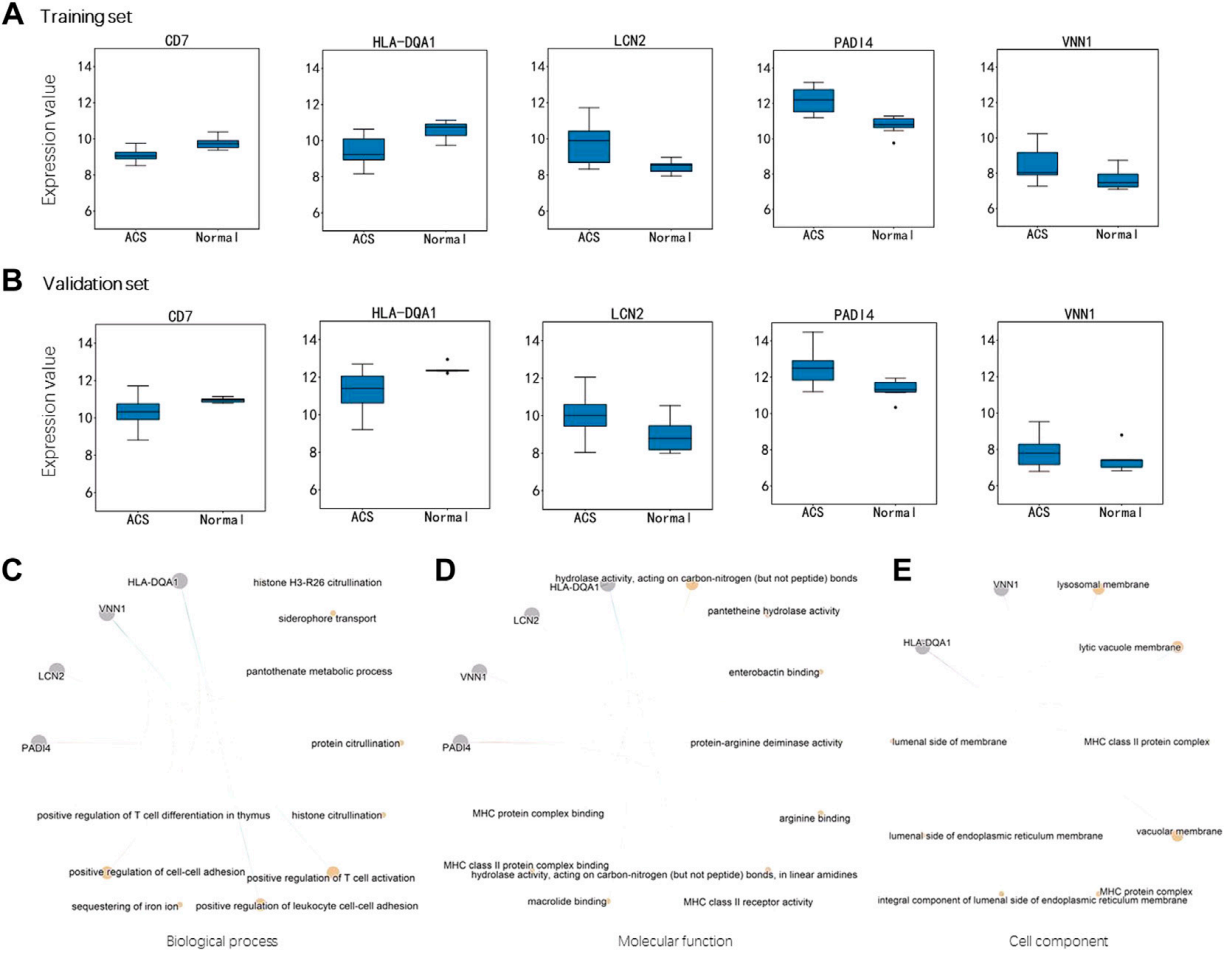
Expression difference of the genes in miSig **(A)** Expression difference of the genes in the training set. **(B)** Expression difference of the genes in the validation set. Enriched functions of the miSig genes in the ontology of Biological Process **(C)**, Molecular Function **(D)**, and Cell component **(E)**.

In the training set, CD7 and HLA-DQA1 were down-regulated in ACS group while LCN2, PADI4 and VNN1 were up-regulated (Figure 5A). The same trend was also observed in the validation set (Figure 5B). Interestingly, the imSig genes in the normal group generally expressed in a small range, whereas they fluctuated in expression among the ACS samples. Comparing to the DEIGs, the imSig genes were implemented in some specific functions, such as positive regulation of leukocyte cell-cell adhesion, positive regulation of T cell activation, ect. PADI4 functions in histone and protein citrullination (Figure 5C), which increased innate immunostimulatory capacity, and immune complexes containing citrullinated histones activated macrophage cytokine production and propagated neutrophil activation. As mentioned above, accordingly, PADI4 is involved in granulocyte and macrophage development causing inflammation and immune response. For the ontologies of molecular functions and cellular component, the imSig genes are enriched in enterobactin binding and pantetheine hydrolase activity (Figure 5D) and tend to resident in lysosomal and vacuolar membrane (Figure 5E).

## Discussion

In this work, we developed an ACS prediction signature based on the expression of iron metabolism-related genes and identified novel serum iron gene markers for early stage of ACS. Five genes, PADI4, HLA-DQA1, LCN2, CD7, and VNN1, were calculated using Elastic Net and included in the final model imSig, which outperforms the other machine learning methods in the validation set.

It has been reported that iron is essential for numerous biological processes, such as oxygen and lipid metabolism, protein production, cellular respiration, and DNA synthesis. In addition to the physiological role, disorders of iron metabolism are also involved in the pathological mechanisms of several common human diseases, such as type 2 diabetes, obesity, non-alcoholic fatty liver disease and coronary artery disease, even participating in the regulation of nerve and brain function. Genetically instrumented serum iron was reported positively associated with type 2 diabetes [ (Wang et al., 2021)]. A meta-analysis indicated that elevated serum ferritin was a risk factors for type 2 diabetes, and soluble transferrin receptor-to-ferritin ratio was inversely related to the risk of type 2 diabetes [ (Liu et al., 2020b)]. Moreover, iron deficiency (ID) is particularly frequent in obese patients due to increased circulating levels of acute-phase reactant hepcidin and adiposity-associated inflammation which reduced iron absorption [ (Bjørklund et al., 2021)]. Jordi et al. uncovered microbiome- and iron-linked metabolomic and transcriptomic signatures involving imbalances in gluconeogenic metabolites, ketone bodies and cellular transport, which altogether modulate liver fat accumulation [(Mayneris-Perxachs et al., 2021)]. Moreover, blood iron level has been reported have important effects on brain and cognitive function [ (Wenger et al., 2022)] and the change of brain function will also affect the heart disease *vice versa*. Myocardial ischemia can be induced by mental stress and coronary heart disease patients have not only pathological changes of circulation of blood but also abnormal behaviors in their spiritual consciousness [(Geng and Yin, 2017), (Geng, 2022)].

Iron and iron deficiency are increasingly being studied in patients with CVD and accumulating evidences suggest that iron deficiency is associated with high risk of CVD [ (Stoltzfus, 2001; Kang et al., 2012; Das De et al., 2015), (Lamas et al., 2013), (Grammer et al., 2014), (Jankowska et al., 2015), (Lewis et al., 2017), (von Haehling et al., 2015)]. The bivalent ferrous form (Fe2+) can donate electrons whereas the trivalent ferric form (Fe3+) can accept electrons, which are required for oxygen transport and enabled iron deficiency induce CVD [ (von Haehling et al., 2015)]. Meng et al. observed that decreases in the levels of irons in the peripheral blood could be a predictive biomarker of coronary atherosclerosis from a study of 4,243 patients, which is consistent with our results [ (Meng et al., 2022)]. Therefore, serum iron metabolism is worthy of paying more attention to study and elucidate mechanisms of iron homeostasis because of its double-edged impacts.

Ferroptosis is a new form of regulated cell death characterized by iron-dependent lipid peroxidation and involved in many metabolic processes, including iron, lipid and glutathione metabolism (Fang et al., 2022). Iron plays an essential role in inducing ferroptosis, because iron is associated with energy metabolism, which is closely linked to ferroptosis. The role of the identified imSig genes in Ferroptosis will be further investigated in our future work.

The major limitation of our study is the sample size of the training and test set. Merely dozens of samples were used for the construction of the prediction model. However, the datasets used in this study are the only available public resources. To address this problem, we build a cohort study Young-COSMOS including 206 samples to study ACS and non-ACS patients, which is a great complement for the repository of ACS and CAD transcriptome data.

Another problem needs to be addressed is the prediction of ACS subtypes. The root cause is still the problem of sample size. Although several subtypes were included in some dataset, each subtype contains few samples, due to the limited total sample size. In Young-COSMOS, ACS contains three subtypes, i.e., ST-elevation myocardial infarction (STEMI), Non-ST-elevation MI (NSTEMI) and unstable angina (UA), where the size is much larger than the dataset used in the current study. Therefore, we believe an updated and more powerful prediction model will be launched soon.

In conclusion, we found ACS patients have decreased serum iron concentration and constructed a prediction model based on iron metabolism-related genes, which may assist in the early diagnosis of ACS.

## Data Availability

The datasets presented in this study can be found in online repositories. The names of the repository/repositories and accession number(s) can be found in the article/supplementary material.
